# Digital Recruitment and Acceptance of a Stepwise Model to Prevent Chronic Disease in the Danish Primary Care Sector: Cross-Sectional Study

**DOI:** 10.2196/11658

**Published:** 2019-01-21

**Authors:** Lars Bruun Larsen, Jens Sondergaard, Janus Laust Thomsen, Anders Halling, Anders Larrabee Sønderlund, Jeanette Reffstrup Christensen, Trine Thilsing

**Affiliations:** 1 Research Unit of General Practice Institute of Public Health University of Southern Denmark Odense Denmark; 2 Research Unit for General Practice Department of Clinical Medicine Aalborg University Aalborg Denmark; 3 Department of Clinical Sciences in Malmö Centre for Primary Health Care Research Lund University Lund Sweden

**Keywords:** promotion of health, clinical decision support systems, cross-sectional studies

## Abstract

**Background:**

During recent years, stepwise approaches to health checks have been advanced as an alternative to general health checks. In 2013, we set up the Early Detection and Prevention project (Tidlig Opsporing og Forebyggelse, TOF) to develop a stepwise approach aimed at patients at high or moderate risk of a chronic disease. A novel feature was the use of a personal digital mailbox for recruiting participants. A personal digital mailbox is a secure digital mailbox provided by the Danish public authorities. Apart from being both safe and secure, it is a low-cost, quick, and easy way to reach Danish residents.

**Objective:**

In this study we analyze the association between the rates of acceptance of 2 digital invitations sent to a personal digital mailbox and the sociodemographic determinants, medical treatment, and health care usage in a stepwise primary care model for the prevention of chronic diseases.

**Methods:**

We conducted a cross-sectional analysis of the rates of acceptance of 2 digital invitations sent to randomly selected residents born between 1957 and 1986 and residing in 2 Danish municipalities. The outcome was acceptance of the 2 digital invitations. Statistical associations were determined by Poisson regression. Data-driven chi-square automatic interaction detection method was used to generate a decision tree analysis, predicting acceptance of the digital invitations.

**Results:**

A total of 8814 patients received an invitation in their digital mailbox from 47 general practitioners. A total of 40.22% (3545/8814) accepted the first digital invitation, and 30.19 % (2661/8814) accepted both digital invitations. The rates of acceptance of both digital invitations were higher among women, older patients, patients of higher socioeconomic status, and patients not diagnosed with or being treated for diabetes mellitus, chronic obstructive pulmonary disease, or cardiovascular disease.

**Conclusions:**

To our knowledge, this is the first study to report on the rates of acceptance of digital invitations to participate in a stepwise model for prevention of chronic diseases. More studies of digital invitations are needed to determine if the acceptance rates seen in this study should be expected from future studies as well. Similarly, more research is needed to determine whether a multimodal recruitment approach, including digital invitations to personal digital mailboxes will reach hard-to-reach subpopulations more effectively than digital invitations only.

## Introduction

### Background

General health checks are seen as one way to mitigate the rising prevalence of chronic diseases such as cardiovascular disease (CVD), type 2 diabetes mellitus (T2DM), and chronic obstructive pulmonary disease (COPD). Consequently, periodical general health checks are provided to citizens by various national health care systems around the world, including those in the United States, South Korea, Australia, and Germany. However, general health checks have not only failed to show population health effects on CVD and total mortality but may have also widen health inequalities [1-6]. This is probably because of the generally higher uptake of health check initiatives in the populations who are likely to benefit the least—including most notably women and patients of higher age, better health, and higher socioeconomic status (SES). Population-level uptake seems to be determined by an interrelationship between individual and societal facilitators and barriers as well as self-selection [7,8].

During recent years, stepwise approaches to health checks have been advanced as an alternative to general health checks [9,10]. Stepwise approaches usually comprise a risk assessment to identify the at-risk population, followed by health checks tailored to this population. If deemed necessary, behavior change interventions, preventive medical treatment, or a combination of the two may also be included. Various stepwise approaches to health checks have been tested in research studies; however, no long-term effects have been reported [11-15]. In the Danish health care system, health checks are provided to the general population on an opportunistic, nonperiodic, and nontargeted basis. On the basis of a technical feasibility study from 2012 [16], we set up the Tidlig Opsporing og Forebyggelse (TOF; early detection and prevention) project in a partnership with the general practitioners’ (GPs) organization and 10 municipalities of the Region of Southern Denmark [17]. Over a period of 2 years, we developed a stepwise model for systematic and targeted prevention of chronic diseases to be used in the Danish primary care sector. The intervention consisted of a joint intervention and a targeted intervention. The joint intervention was applied to the entire study population in the form of a personal digital health profile. The targeted intervention was only applied to patients who were deemed to potentially benefit from either a health check at their GP or lifestyle coaching provided by the municipal health center. Patients at high risk of a chronic disease were identified using validated risk algorithms for COPD, T2DM, and CVD and were offered a health check at their GP in the form of a medical examination and a health dialogue. Patients with health risk behavior included patients who were not at high risk as determined by the risk algorithms but who engaged in one or more health-risk behaviors such as smoking, high-risk alcohol consumption, poor dietary habits, sedentary behavior, and/or a body mass index above 35. This cohort was offered a short 15-min telephone-based health dialogue with a health professional from the municipal health center. For patients with limited health capabilities, the initial telephone-based health dialogue could be followed up by a 1-hour face-to-face health dialogue. If deemed necessary, the targeted intervention would be complemented by further behavior change intervention or preventive medical treatment. Patients already diagnosed with hypertension, hypercholesterolemia, T2DM, CVD, or COPD by the GP, or who displayed no health risk behaviors were only offered the joint intervention. In line with the Medical Research Council’s recommendations for complex interventions, we tested the acceptability, feasibility, and short-term effects of the intervention in a pilot study in 2 municipalities between April and December 2016 [18].

A novel feature of the pilot study was the use of a personal digital mailbox for recruiting participants. A personal digital mailbox is a secure digital mailbox provided by the Danish public authorities. It is accessed either via a webpage or an app developed for all major operating systems. The digital mailbox is secured by a national 2-phased log-in system (NemID) and is used by all public authorities as well as an increasing number of private companies such as banks and insurance companies. Beyond being both safe and secure, it is a low-cost, quick, and easy way to reach Danish residents [19]. Permanent residents of Denmark are obliged by law to have a digital mailbox and are expected to check it regularly. Short message service text message and mail reminders are optional. Opting out is only possible in special cases, mainly in the event of low information and technology literacy (usually age-related) or cognitive impairment. A total of 90% of the entire population in Denmark and 95% of the target population have a digital mailbox (May 2016) [20].

### Objective

This study reports on the association between the rates of acceptance of 2 digital invitations sent to a personal digital mailbox and sociodemographic determinants, medical treatment, and health care usage in a stepwise primary care model for the prevention of chronic diseases.

## Methods

### Design

We conducted a cross-sectional analysis of the rates of acceptance of 2 digital invitations sent to residents from 2 Danish municipalities randomly selected to take part in a pilot study of the TOF project (NCT02797392).

### Population

The target population consisted of citizens born between 1957 and 1986 and residing in Haderslev or Varde, 2 rural municipalities located in the southern part of Jutland, Denmark. The population of both municipalities totals 106,081 citizens (2015).

### Setting

The Danish health care system comprises a strong, publicly funded primary care sector, which includes municipal health centers and GP clinics [21]. GPs operate a patient list system, with an average of 1600 patients per GP. On average, 2 GPs work in a given clinic. Municipal health centers serve the entire population with primary prevention such as smoking cessation and dietary advice, whereas GPs manage and coordinate secondary prevention, including treatment for hypertension, hypercholesterolemia, and diabetes.

### Recruitment Procedure

In January 2016, all 66 GPs residing in the municipalities of Varde and Haderslev received a written invitation, with an enclosed project agreement form and a prepaid return envelope. Nonresponse was followed up by a telephone call to the GP. Using the Regional Primary Care Administrative System (KMD Sygesikring), the regional health authorities identified a source population of 200 randomly selected patients extracted from each of the participating GPs’ patient lists. From the source population, we excluded patients having either no digital mailbox or residing outside the municipalities of Varde or Haderslev.

Participants were recruited using 2 digital invitations sent to their personal digital mailbox (Multimedia Appendix 1). Both digital invitations consisted of a 1-page PDF file in Danish and included a highly visible hyperlink to a Web-based digital support system, on which participants would provide both consent and access to the personal digital health profile [22]. The first digital invitation was sent out in April 2016, with the aim of obtaining consent to participate in the study and to access specific information from their GP’s electronic patient record (EPR) system, including International Classification of Primary Care, 2nd edition) codes for diagnoses and anatomical therapeutic chemical (ATC) codes for medical prescriptions. The second digital invitation was aimed at providing participants with a digital health profile. This invitation was sent out in September 2016 to participating patients who were registered with the same GP as when they consented and who still resided in the municipalities of Varde or Haderslev. A nonresponse triggered up to 2 reminders 1 week apart.

### Outcomes

In this paper, we report on both the consent to take part in the study and the uptake of the personal digital health profile. The main outcome relates to the acceptance of the first digital invitation and is operationalized in terms of consent or nonconsent to take part in the study. Consent is defined as the provision of informed consent to participate in the study; nonconsent includes both nonresponse and active nonconsent. The second outcome relates to the acceptance of the second digital invitation and is operationalized in terms of uptake or nonuptake of the personal digital health profile. Uptake is defined as patients who gave their active consent and received a personal digital health profile. The results from the second digital invitation are presented in Multimedia Appendices 2 and 3.

### Variables

Registry variables for the entire study population were retrieved from the administrative registry and Statistics Denmark and linked with the patients’ Danish Personal Identification numbers. EPR information was retrieved directly from participating GPs’ EPR systems and related purely to consenting patients (Table 1). All participants were pseudonymized when linking project data and national registers from Statistics Denmark.

Age was categorized in 10-year age groups. Country of origin was retrieved for the year 2016 and categorized as Danish, Western, or non-Western origin. Cohabitational status was retrieved for the year 2016 and categorized as cohabiting or single. Highest attainable educational level was retrieved for October 2015 and categorized as secondary school, high school, vocational education, or higher education. Occupation was retrieved for November 2014 and categorized according to the Organisation for Economic Co-operation and Development equivalence scales into 5 groups: employed, self-employed, unemployed or on benefits, social welfare recipients, or other [30]. The distinction between unemployment benefits and social welfare is that unemployment benefits are accessible to citizens who have been unemployed for less than 2 years and who are members of a voluntary unemployment benefit fund. Social welfare benefits are for all other unemployed persons who can take up a job. Others represent, for example, nonworking persons from a family that relies on 1 income only. Family income was retrieved for 2013, 2014, and 2015, defined by the mean annual net income of the household, and categorized into quartiles. “Partner in project” describes whether your partner (if cohabiting) participated as well. Partner in project is categorized in a binary yes or no variable.

Information on prescriptions and diagnoses was combined as a proxy for medical treatment (Table 2). Prescriptions were retrieved for the period from May 2014 to April 2016 as ATC codes. We chose a 2-year period, as prescriptions may be filed up to 2 years after their date of issue. International Classification of Diseases 10th edition (ICD-10) codes were retrieved for the period from January 2013 to April 2016. Medical treatment was defined as either registered with an ATC code, ICD-10 code, or both during the periods specified above.

**Table 1:** Analyses of associations between patient characteristics and acceptance.

Variable		First digital invitation	Second digital invitation	
Presentation of results		Results section	Multimedia Appendix 2	Multimedia Appendix 3
Denominator		Study population	Study population	Consent to the first digital invitation
Outcome variable		Consent or nonconsent to the first digital invitation	Uptake or nonuptake of the second digital invitation	Uptake or nonuptake of the second digital invitation
**Exposures**				
	Sociodemographics	Age^a,b^	Age^a,b^	Age^a,b^
		Sex^a,b^	Sex^a,b^	Sex^a,b^
		Country of origin^b^	Country of origin	Country of origin
		Highest educational attainment^c^	Highest educational attainment^c^	Highest educational attainment^c^
		Occupational status^d^	Occupational status^d^	Occupational status^d^
		Family income^e^	Family income^e^	Family income^e^
		Cohabitational status^b^	Cohabitational status^b^	Cohabitational status^b^
		Partner consent^f^	Partner consent^f^	Partner consent^f^
	Medical treatment	Prescriptions from primary care or hospitals (anatomical therapeutic chemical [ATC] codes)^g^	Prescriptions from primary care or hospitals (ATC codes)^g^	Prescriptions from primary care or hospitals (ATC codes)^g^
		Hospital discharge diagnoses (International Classification of Diseases 10^th^ edition [ICD-10] codes)^h^	Hospital discharge diagnoses (ICD-10 codes)^h^	Hospital discharge diagnoses (ICD-10 codes)^h^
	Health care usage	Administrative primary care codes^i,j^	Administrative primary care codes^i,j^	Administrative primary care codes^i,j^; health checks^i,j^

^a^Danish National Administrative Primary Care System (Praksys).

^b^Danish Civil Registration System [23].

^c^Danish Education Register [24].

^d^Danish Registers of Labour Market Affiliation [25].

^e^Danish Registers of Personal Income and Transfer Payments [26].

^f^Questionnaire data.

^g^Danish National Prescription Registry [27].

^h^Danish National Patient Registry [28].

^i^General practitioners’ electronic patient record.

^j^Danish National Health Service Register [29].

**Table 2:** International Classification of Diseases 10th edition codes and anatomical therapeutic chemical classification codes used to define when a medical condition of chronic obstructive pulmonary disease, cardiovascular disease, or diabetes mellitus had been registered.

Medical condition	ICD-10^a^ codes registered from January 2013 to April 2016	ATC^b^ therapeutic codes for prescribed medicine registered from May 2014 to April 2016
COPD^c^	J44	R03AC18, R03AC19, R03AL03, R03AL04, R03AL05, R03BB04, R03BB05, and R03BB06
CVD^d^	I1-I7 and E78 (except: I0, I16, I60, I73, and I78)	C (except: C01CA and C05)
Diabetes mellitus	E10-E13	A10

^a^ICD-10: International Classification of Diseases 10th edition.

^b^ATC: anatomical therapeutic chemical.

^c^COPD: chronic obstructive pulmonary disease.

^d^CVD: cardiovascular disease.

Health care usage was determined from administrative codes registered by the GP and retrieved for the period from May 2013 to April 2016. To this end, we also examined EPR information on laboratory test results. Administrative codes were used to extract frequent GP attenders, GP attenders, and usage of specific administrative codes pertaining to laboratory tests and preventive consultations. Frequent attenders referred to the top 10% of patients, who on average contacted the GP the most—either in person or by phone—during the 3 years from May 2013 to April 2016 [31]. GP attenders were defined as patients having contacted their GP during the 2-year period from May 2014 to April 2016. Laboratory tests comprised blood samples (administrative codes 2101 and 2601), peak flow tests (7183), spirometries (7113), and home blood pressure monitoring (2146) during the period from May 2014 to April 2016. Furthermore, we retrieved information on specific preventive consultations (0120)—that is, special consultations for coaching patients with health risk behaviors and patients diagnosed with a chronic disease. To determine if a patient had received a preventive health check within a period of 2 years before consenting to the study (May 2014 to May 2016), we retrieved blood pressure (systolic and diastolic blood pressure), lung function (forced expiratory volume [FEV_1_], forced vital capacity [FVC], and FEV_1_/FVC), glycated hemoglobin, and lipids (total cholesterol, high-density lipoprotein, and low-density lipoprotein) measurements from the GPs’ EPR systems. A health check was defined as having had 2 or more of the above-mentioned values measured in the same consultation.

### Analysis

Statistical associations are presented as crude figures, age- and sex-adjusted figures, and as models minimally adjusted for known confounders. Poisson regression with robust variance error was used rather than logistic regression to obtain incidence rate ratios (IRR). The binary outcome variable of consent or nonconsent was interpreted as a continuous variable with the only counts being 0 or 1. The minimally adjusted model was developed from a causal direct acyclic graph (DAG), built on the current evidence of the determinants of attendance at health checks [32]. Attendance at health checks was the outcome variable of the DAG. Significance level was set at *P*<.05.

A data-driven chi-square automatic interaction detection (CHAID) method was used to generate a decision tree analysis to identify interactions and a hierarchy of variables predicting the chosen outcome variable (root node) [33]. Using chi-square tests of interdependence, a CHAID analysis clusters categories within each predictor variable to determine what predictor variables are associated with the outcome. Subsequently, the predictor variables associated with the outcome are ordered hierarchically. The specific variable order is determined by the Bonferroni *P* value of each variable such that the predictor variable with the smallest *P* value (strongest association) is placed at the top of the hierarchy (parent node). The minimum number of observations for each split in the decision tree was set at 500 (child node) and 200 for each node (terminal node). In the analysis of the acceptance of the second digital invitation, each split in the decision tree was set to 100 (child node) and 20 for each node (terminal node) because of the limited number of observations.

Statistical analysis was performed on secure servers at Statistics Denmark using Stata 14 (Statacorp).

### Ethics Approval and Consent to Participate

The study was approved by the Danish Data Protection Agency (J.Number 2015-57-0008) and registered at Clinical Trial Gov (Unique Protocol ID: TOFpilot2016). According to Danish regulations (Act on Research Ethics Review of Health Research Projects [section 14,2]), this study does not need approval from a health research ethics committee as no research on human tissue or other biological material is performed. The study complies with the Helsinki Declaration, with informed consent to study participation and to disclosure of data from the GPs’ EPR obtained from all participants.

## Results

### Recruitment and Overall Uptake

**Figure 1:**
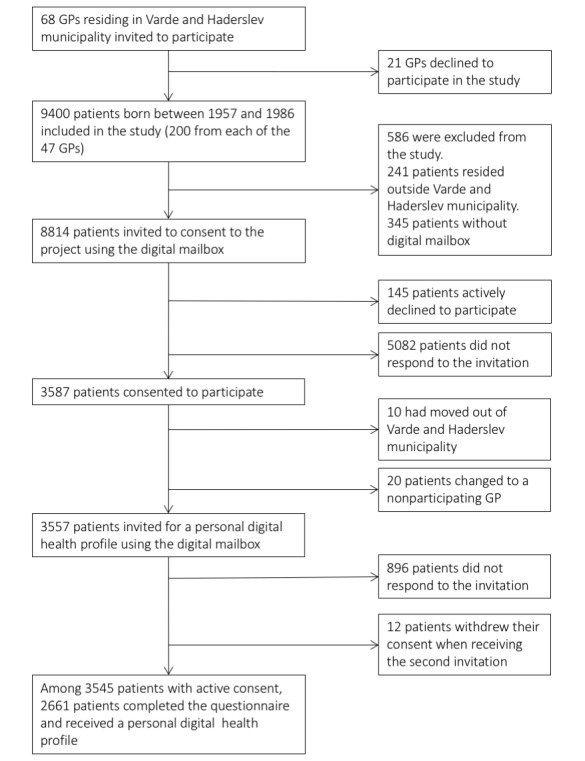
Flow diagram from source population to study population. GP: general practitioner.

Of the 68 GPs residing in the 2 municipalities, 47 GPs from 18 clinics agreed to participate in the study (Figure 1). This provided us with a source population of 9400 patients. However, a total of 586 patients did not meet the inclusion criteria, which is why only 8814 received the first invitation. Initially, a total of 3587 patients consented to participate, but among them, 30 patients moved from the municipality to a nonparticipating GP, and 12 withdrew their consent after receiving the second invitation. This resulted in 3545 active consenters from the first round of invitations (Multimedia Appendix 2). Of the patients who accepted the first digital invitation (n=3545), 75.06% (2661/3545) also accepted the second digital invitation (Multimedia Appendix 3; Table 3).

**Table 3:** Descriptive analysis of determinants of the acceptance of the first digital invitation.

Determinants			Consenters (N=3545), n (%)	Nonconsenters (N=5269), n (%)	Total (N=8814), n (%)	Missing, n (%)
**Demography^a^**						
	**Age (years)**					
		29-39	732 (20.64)	1921 (36.45)	2653 (30.09)	0 (0.00)
		40-49	1151 (32.46)	1875 (35.58)	3026 (34.33)	0 (0.00)
		50-60	1662 (46.88)	1473 (27.95)	3135 (35.56)	0 (0.00)
	**Sex**					
		Male	1590 (44.85)	2845 (53.99)	4379 (49.68)	0 (0.00)
		Female	1955 (55.14)	2424 (46.00)	4435 (50.31)	0 (0.00)
	**Country of origin**					
		Denmark	3385 (95.48)	4446 (84.38)	7831 (88.44)	18 (0.20)
		Western	91 (2.56)	458 (8.69)	549 (6.22)	18 (0.20)
		Non-Western	69 (1.94)	347 (6.58)	416 (4.71)	18 (0.20)
	**Cohabitation**					
		Single	726 (20.47)	1516 (28.77)	2242 (25.43)	18 (0.20)
		Cohabiting	2819 (79.52)	3735 (70.88)	6554 (74.35)	18 (0.20)
	**Partner in project**					
		Yes	2037 (57.46)	2715 (51.52)	4752 (53.91)	18 (0.20)
		No	1508 (42.53)	2536 (48.13)	4044 (45.88)	18 (0.20)
**Socioeconomy**						
	**Educational attainment**					
		Secondary school	513 (14.47)	1194 (22.66)	1707 (19.36)	583 (6.61)
		High school	143 (4.103)	213 (4.04)	356 (4.03)	583 (6.61)
		Vocational education	1604 (45.24)	2199 (41.73)	3803 (46.14)	583 (6.61)
		Higher education	1216 (34.30)	1149 (21.80)	2365 (26.83)	583 (6.61)
	**Employment status**					
		Employed	2891 (81.55)	3719 (70.58)	6610 (74.99)	105 (1.19)
		Self-employed	170 (4.79)	260 (4.93)	430 (4.87)	105 (1.19)
		Benefits	88 (2.48)	184 (3.49)	272 (3.08)	105 (1.19)
		Social welfare	340 (9.59)	806 (15.29)	1146 (13.00)	105 (1.19)
		Other	51 (1.43)	200 (3.79)	251 (2.84)	105 (1.19)
	**Family income**					
		Low	559 (15.76)	1488 (28.24)	2047 (23.22)	130 (1.47)
		Middle-low	806 (22.73)	1341 (25.45)	2147 (24.35)	130 (1.47)
		Middle-high	989 (27.89)	1246 (24.64)	2235 (25.35)	130 (1.47)
		High	1183 (33.4)	1072 (20.34)	2255 (25.58)	130 (1.47)
**Medical treatment^b^**						
	**Prescriptions and diagnoses**					
		Treatment	763 (21.52)	973 (18.46)	1736 (19.69)	0 (0.00)
		No treatment	2782 (78.47)	4296 (81.53)	7078 (80.30)	0 (0.00)
**Health care usage^c^**						
	**Attendance to general practitioner (GP)**					
		Yes	3173 (89.50)	4372 (82.97)	7545 (85.60)	0 (0.00)
		No	372 (10.49)	897 (17.02)	1269 (14.39)	0 (0.00)
	**Frequent attender**					
		Yes	368 (10.38)	584 (11.08)	952 (10.80)	0 (0.00)
		No	3177 (89.61)	4685 (88.91)	7862 (89.19)	0 (0.00)
	**Laboratory tests at GP**					
		Yes	2053 (57.91)	2471 (46.89)	4524 (51.32)	0 (0.00)
		No	1492 (42.08)	2798 (53.10)	4290 (48.67)	0 (0.00)
	**Preventive consultation at GP**					
		Yes	431 (12.15)	516 (9.79)	947 (10.74)	0 (0.00)
		No	3114 (87.84)	4753 (90.20)	7867 (89.25)	0 (0.00)

^a^Social registries.

^b^Anatomical therapeutic chemical codes and International Classification of Diseases 10th edition codes related to diabetes, cardiovascular disease, and chronic obstructive pulmonary disease.

^c^Administrative codes from the general practitioner.

### Acceptance of the First Digital Invitation

The Poisson regressions showed that a higher rate of acceptance of the first digital invitation was associated with sociodemographic factors, including higher age, income, and educational attainment (Table 4). A higher rate of acceptance was also associated with being female, employed, born in Denmark, and cohabiting. Patients not diagnosed with or in treatment for T2DM, CVD, or COPD were more likely to accept the first digital invitation than patients in treatment. Similarly, the acceptance rate was higher among patients who had seen their GP or who had registered 1 or more laboratory tests at their GP within 2 years of giving consent. We found no association between the likelihood of accepting the first digital invitation and the frequency of GP appointments, being registered with a preventive consultation, or having a partner that also consented to the study.

**Figure 2:**
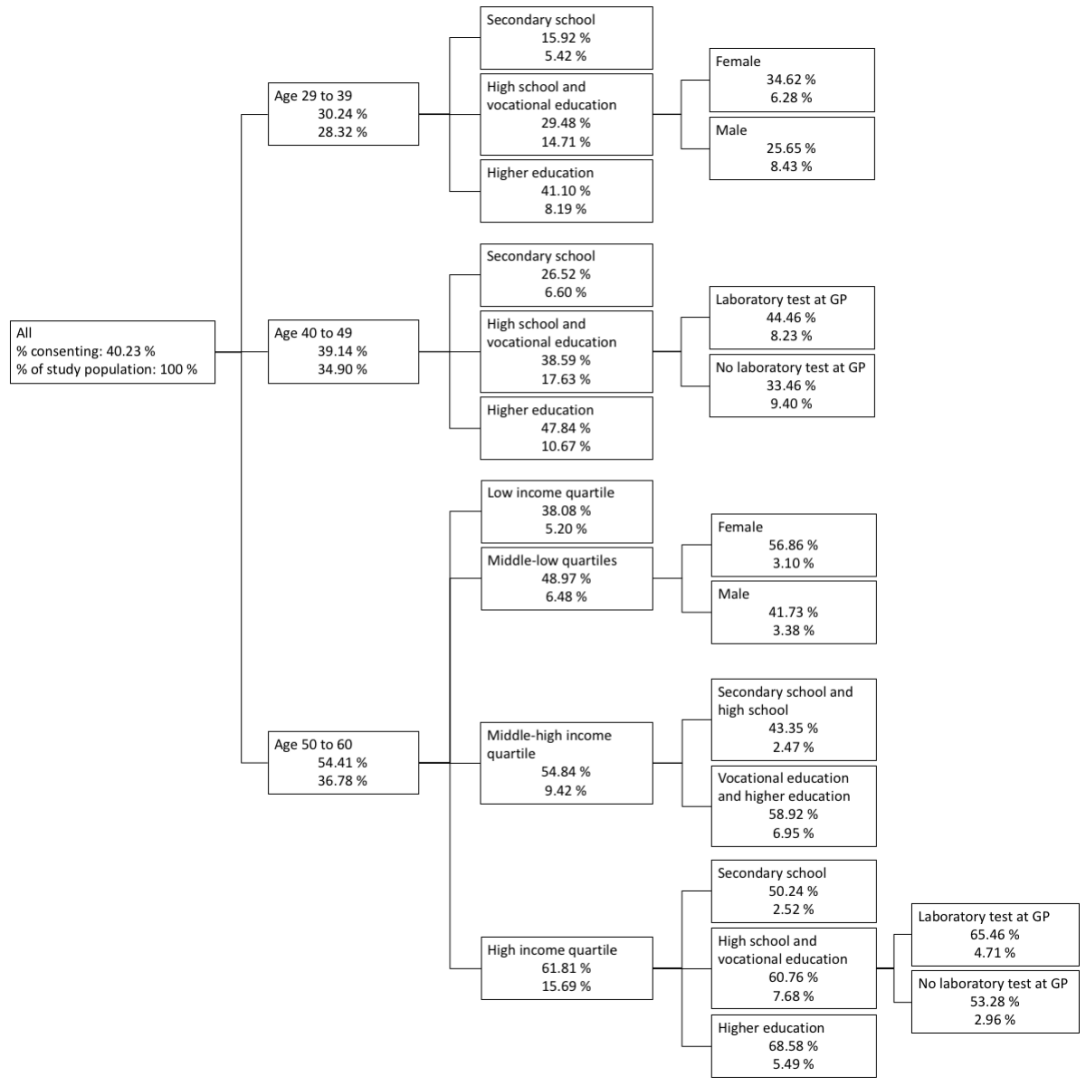
Chi-square automatic interaction detection analysis of the acceptance of the first digital invitation. GP: general practitioner.

The CHAID analysis showed that age was the strongest predictor of accepting the first digital invitation followed by the educational attainment in patients below the age of 50 years and income in patients above the age of 50 years (Figure 2). The CHAID showed large subgroup differences in acceptance rates. Of patients below the age of 40 years, with secondary school as the highest educational attainment, 15.92% accepted the first digital invitation. By contrast, the acceptance rate among patients above the age of 50 years, with high income and with at least a bachelor-level education was 68.58 %.

**Table 4:** Analysis of associations between acceptance of the first digital invitation and sociodemographic determinants, medical treatment, and health care usage.

Determinants		Sample size (N)	Model 1 (crude)		Model 2 (adjusted for age and sex)		Model 3 (minimally adjusted)	
			IRR^a^ (95% CI)	*P* value	IRR (95% CI)	*P* value	IRR (95% CI)	*P* value
**Age^b^** **(years)**								
	29-39	2653	1 (0)	—^c^	1 (0)	—	1 (0)	—
	40-49	3026	1.08 (1.06-1.10)	.001	1.08 (1.06-1.10)	.001	1.08 (1.06-1.10)	.001
	50-60	3135	1.20 (1.18-1.22)	.001	1.20 (1.18-1.22)	.001	1.20 (1.18-1.22)	.001
**Sex^b^**								
	Female	4435	1 (0)	—	1 (0)	—	1 (0)	—
	Male	4379	0.94 (0.93-0.95)	.001	0.94 (0.93-0.95)	.001	0.94 (0.93-0.95)	.001
**Country of origin^b^**								
	Denmark	7831	1 (0)	—	1 (0)	—	1 (0)	—
	Western	549	0.81 (0.79-0.84)	.001	0.84 (0.81-0.86)	.001	0.81 (0.79-0.84)	.001
	Non-Western	416	0.81 (0.79-0.84)	.001	0.84 (0.81-0.86)	.001	0.81 (0.79-0.84)	.001
**Cohabitation^d^**								
	Single	2242	1 (0)	—	1 (0)	—	1 (0)	—
	Cohabiting	6554	1.08 (1.06-01.10)	.001	1.07 (1.05-01.08)	.001	1.05 (1.03-01.07)	.001
**Partner in project^e^**								
	Yes	4752	1 (0)	—	1 (0)	—	1 (0)	—
	No	4044	0.96 (0.95-0.98)	.001	0.96 (0.95-0.98)	.001	1.00 (0.99-1.02)	.70
**Educational attainment^f^**								
	Secondary school	1707	1 (0)	—	1 (0)	—	1 (0)	—
	High school	356	1.08 (1.04-1.12)	.001	1.09 (1.05-1.13)	.001	1.09 (1.05-1.14)	.001
	Vocational education	3803	1.09 (1.07-1.12)	.001	1.10 (1.08-1.12)	.001	1.10 (1.08-1.12)	.001
	Higher education	2365	1.16 (1.14-1.19)	.001	1.17 (1.14-1.19)	.001	1.16 (1.14-1.19)	.001
**Employment status^d^**								
	Employed	6610	1 (0)	—	1 (0)	—	1 (0)	—
	Self-employed	430	0.97 (0.94-1.00)	.089	0.96 (0.93-1.00)	.03	0.97 (0.94-1.00)	.05
	Benefits	272	0.92 (0.88-0.96)	.001	0.93 (0.89-0.97)	.001	0.95 (0.91-1.00)	.03
	Social welfare	1146	0.90 (0.88-0.92)	.001	0.90 (0.88-0.92)	.001	0.94 (0.92-0.96)	.001
	Other	251	0.84 (0.80-0.87)	.001	0.85 (0.82-0.89)	.001	0.92 (0.87-0.97)	.005
**Family income^d^**								
	Low	2047	1 (0)	—	1 (0)	—	1 (0)	—
	Middle-low	2147	1.08 (1.06-1.10)	.001	1.07 (1.05-1.10)	.001	1.05 (1.02-1.07)	.001
	Middle-high	2235	1.13 (1.11-1.16)	.001	1.11 (1.09-1.14)	.001	1.06 (1.04-1.09)	.001
	High	2255	1.20 (1.17-1.22)	.001	1.14 (1.12-1.17)	.001	1.08 (1.05-1.10)	.001
**Prescriptions and diagnoses^d^**								
	Treatment	1736	1 (0)	—	1 (0)	—	1 (0)	—
	No treatment	7078	0.97 (0.95-0.99)	.001	1.02 (1.00-1.04)	.02	1.02 (1.00-1.04)	.04
**Attendance at general practitioner (GP)^d^**								
	Yes	7545	1 (0)	—	1 (0)	—	1 (0)	—
	No	1269	0.91 (0.89-0.93)	.001	0.93 (0.91-0.95)	.001	0.95 (0.93-0.98)	.001
**Frequent attender to GP^d^**								
	Yes	952	1 (0)	—	1 (0)	—	1 (0)	—
	No	7862	1.01 (0.99-1.04)	.30	1.03 (1.01-1.06)	.007	1.02 (1.00-1.05)	.07
**Laboratory tests at GP^d^**								
	Yes	4524	1 (0)	—	1 (0)	—	1 (0)	—
	No	4290	0.93 (0.91-0.94)	.001	0.95 (0.94-0.97)	.001	0.95 (0.94-0.97)	.001
**Preventive consultation at GP^d^**								
	Yes	947	1 (0)	—	1 (0)	—	1 (0)	—
	No	7867	0.96 (0.94-0.98)	.001	1.00 (0.98-1.02)	.94	0.99 (0.97-1.02)	.66

^a^Incidence rate ratio.

^b^Model 3 adjustments: no adjustments.

^c^Reference category.

^d^Model 3 adjustments: age, sex, country of origin, and education.

^e^Model 3 adjustments: cohabitation.

^f^Model 3 adjustments: age, sex, and country of origin.

### Acceptance of the Second Digital Invitation

In the entire study population, the rate of acceptance of the second digital invitation showed associations similar to the ones in the analysis of the first digital invitation. The only differences were higher rates of acceptance among frequent GP attenders and no association with attendance or nonattendance to the GP. The similarity also applied to the CHAID analysis, in which age was shown to be the strongest predictor, followed by educational attainment in the age below 50-years bracket and income in the age above 50-years bracket. The CHAID analysis showed a rate of acceptance of 8.37% in patients below the age of 40, with secondary school as their highest educational attainment and income below 50% of the median. By contrast, among patients aged more than 50 years, with an income of more than 50% above the median and at least a bachelor-level education, 60.40% accepted the second digital invitation.

### Acceptance of the Second Digital Invitation Among Consenters (N=3545)

Among the patients who accepted the first digital invitation, the Poisson regressions indicated associations between the rate of acceptance of the second digital invitation and most sociodemographic variables. The rate of acceptance increased with age, educational attainment, and income. Being female, employed, and born in Denmark also correlated positively with rates of acceptance. We saw higher rates of acceptance among patients who had not seen their GP within 2 years before consenting to the study. No other variables describing health status or health care usage showed an association with rates of acceptance—that includes having received a health check during a period of 2 years before consenting to the study.

The CHAID analysis showed that age was the strongest predictor of acceptance of the second digital invitation among those who accepted the first digital invitation and the only predictor in patients younger than 50 years. In patients older than 50 years, income followed age as the second strongest predictor. Specifically, when it came to the patients older than 50 years, with a middle-low or middle-high income, the CHAID analysis revealed that nonattendance at the GP and not receiving medical treatment during a period of 2 years before consenting were both strong predictors of whether or not participants accepted the second digital invitation.

## Discussion

### Main Findings

In this study, 40.22% of our sample accepted the first digital invitation and 30.19% accepted both digital invitations. That is, among those who accepted the first digital invitation, 75.06% also accepted the second. The rates of acceptance of both digital invitations were higher among women, elderly patients, patients of higher SES, and patients not diagnosed with or in treatment for T2DM, COPD, or CVD. Patients who had seen their GP within the past 2 years were also more likely to accept the digital invitations. The frequency of GP appointments, registering for a preventive consultation, or having a partner who had accepted the first digital invitation showed no association with the acceptance of either invitation. In the subpopulation of patients (N=2661) who accepted the first digital invitation, women, patients of relatively high age and SES, and patients who had not seen their GP for a period of 2 years before giving their consent showed a higher rate of acceptance of the second invitation. No other health care usage, including having had a health check within the previous 2 years or being diagnosed with or in treatment for T2DM, COPD, or CVD showed any association with the rate of acceptance.

Low patient uptake of stepwise models for preventing chronic diseases seems to be the current norm. This trend may be attributed to a combination of a recent overall increase in the use of preventive health checks in primary care and a decrease in response rates to research studies in general [34,35]. A recent Dutch study of a stepwise prevention model showed an uptake rate of 29% in patients aged 45 to 70 years, whereas an Australian study had an initial uptake rate of 31% in patients aged 40 to 64 years [11,36]. A feasibility study to this study showed an uptake rate of 63% using paper-based invitations, with a link to a Web-based questionnaire and an enclosed hard copy questionnaire and return envelope [37]. Two other Danish studies of stepwise models showed uptake rates of 55% in a general population aged 30 to 49 years and 30% in a population of social housing residents aged 45 to 70 years [38,39]. Both Danish studies used a proactive approach by which paper-based invitations indicated a prebooked time and date—a method which has been shown to garner increased response rates in a previous study [40]. Furthermore, the associations between acceptance of digital invitations and socioeconomic determinants are in line with the evidence from other Danish and European studies of health checks [6,38,41]. However, the CHAID analyses showed that the differences in rates of acceptance among the SES groups in our study are larger than those observed in the 2 Danish studies mentioned above [38,39]. The evidence of the association between uptake of stepwise models and medical treatment or health care usage is scarce and largely inconclusive. This is presumably because of a general lack of health and health care information on nonresponders. Nonetheless, the results of medical treatment and health care usage in this study differ somewhat from previous studies. We show slightly higher rates of acceptance of the digital invitations among patients not diagnosed with or in treatment for T2DM, COPD, or CVD. This is in line with a comparable Danish study [38], whereas other studies report either higher uptake among patients with chronic diseases [42,43] or no association [44,45]. The inconsistency in results could be explained by different definitions of medical treatment. Similarly, different definitions of preventive services could explain why we see no association between acceptance of the digital invitations and use of preventive consultations at the GP, whereas other studies suggest an association [38]. We saw no association between acceptance of either invitation and having had a health check during a period of 2 years leading up to consent; however, other studies have consistently found that prior use of health checks seems to increase the likelihood of getting another health check [46-48]. Interestingly, we found an association between not having had a GP appointment during the previous 2 years and a higher rate of acceptance of the second digital invitation among those who also accepted the first digital invitation. This may suggest that true compliers to the study (ie, patients who would not have taken up the offer had they not been invited) are more likely to also accept the second digital invitation than always-users (ie, patients who always respond to invitations to participate in preventive services) [49]. As we saw no association between the rates of acceptance of the second digital invitation and other variables of health care utilization, this result should be interpreted with great care and examined further in future studies.

### Efficacy of Digital Recruitment

To our knowledge, this is the first study to report on the rates of acceptance of digital invitations to participate in a stepwise model for prevention of chronic diseases. Moreover, it is most likely the first to report on digital recruitment to a health intervention using digital invitations. We cannot establish whether rates of acceptance would have been different if recruitment had been paper-based, as we did not include a random subpopulation, which received paper-based invitations. To our knowledge, comparisons of digital and paper-based invitations sent by regular mail have only been reported once in a randomized study by Ebert et al [19]. This study showed that 50- to 59-year-old responders to the digital invitation were more likely to be of higher SES than their counterparts who responded to paper invitations. However, no differences were seen in those aged 30 to 39 years. The overall rate of acceptance of digital invitations and paper-based invitations was comparable. The rate of acceptance of digital invitations in this study and the results reported by Ebert et al may suggest slightly lower overall acceptance rates and slightly larger SES differences when using digital invitations. However, the rate of acceptance of digital invitations combined with Web-based data collection may resemble the emergence of the combined paper-based invitation and Web-based data-collection approach, where the advent of Web-based data collection methods precipitated an initial drop in the rate of acceptance [34].

In addition, digital invitations sent to personal digital mailboxes seem to be an especially suitable and low-cost recruitment method for patients of high SES. It is well known that risk factors of chronic lifestyle-related diseases are clustered in low SES populations [50]. To generate population health effects, stepwise models for the prevention of chronic diseases may have to employ other low-tech recruitment approaches, which complement digital invitations. Results from the health check program of the British National Health Service indicate that uptake may increase over time, with a clear focus on the hardest-to-reach populations [51]. Thus, it would appear that the lower uptake among patients of lower SES found in this as well as in many other studies can be eliminated, or even inverted, by a focused recruitment effort aimed at deprived communities and outreach services [52,53]. However, at present, digital recruitment is only applicable in a few countries, among others the Nordic countries of Denmark, Norway, and Sweden. When digital mail gets more widespread, the results from the described recruitment procedure and intervention may be well applicable in other settings as well.

Nonetheless, more studies of digital invitations are needed to determine if the acceptance rates seen in this study and in the study by Ebert et al could be expected from future studies as well. Similarly, more research is needed to determine whether a multimodal recruitment approach, including digital invitations to personal digital mailboxes will reach other subpopulations more effectively than digital invitations only.

### Strengths and Limitations

The main strengths of this study relate to the high validity of the registries of Statistics Denmark and the random sampling of patients from a large number of GP clinics. Especially, the health and social registries are of high quality, with few missing cases and up-to-date information that has been registered either immediately before study commencement or during another specified period before study commencement [54]. Thus, the impact is most likely negligent because of the strong association between acceptance of the digital invitations and SES. All direct contacts with the GP are most likely both valid and complete as this type of information is automatically registered onto patients’ personal health insurance cards. Other administrative data from the primary care sector on specific tasks performed as part of a consultation, such as taking a blood sample, a spirometry, or having a preventive consultation, may be incomplete and more prone to human error and should as such be interpreted with care. Another strength is the combination of DAG and CHAID analyses to establish a both theory-driven and data-driven analytical approach. The DAG established adjustments to the Poisson regression models based on a theoretical and evidence-based causal model. The CHAID used the data from this study and identified the strongest predictors of attendance. However, residual confounding and collider bias cannot be eliminated in the regressions because of both a rather complex causal model, conditional independence between exposures, and the unavailability of a number of exposures—especially health-risk behaviors as well as cognitive and psychological parameters [6]. Finally, the invitations sent to the digital mailbox and the digital support system were in Danish language only, which may have had a negative impact on the rate of acceptance among people originating from outside of Denmark.

### Conclusions

To our knowledge, this is the first study to report on the rates of acceptance of digital invitations to participate in a stepwise model for prevention of chronic diseases. We show acceptance rates of 40% for a first digital invitation and 30% for a second digital invitation, the rates being higher among women, elderly patients, and patients of higher SES; patients not diagnosed with, or in treatment for T2DM, COPD, and/or CVD; and patients having attended the GP within a period of 2 years before consent. A total of 75% of those who accepted the first digital invitation also accepted the second. On one hand, the 2 digital invitations seem to deepen the sociodemographic differences in acceptance compared with a single digital invitation; on the other hand, patients who had not consulted a GP during a period of 2 years before the study, and who were not receiving medical treatment, showed a significantly higher rate of acceptance of the second invitation when it came to those who had accepted the first digital invitation. This suggests that compliers are more prone to accepting the second digital invitation than always-users are. In future studies, multimodal recruitment approaches, which complement digital invitations, are warranted to increase the rates of acceptance among harder-to-reach subpopulations.

## Appendix

Multimedia Appendix 1 Invitations (in Danish).

Multimedia Appendix 2 Data on the acceptance of the second digital invitation in the entire study population.

Multimedia Appendix 3 Data on the acceptance of the second digital invitation among those who accepted the first digital invitation.

## Abbreviations

ATC: anatomical therapeutic chemical
CHAID: chi-square automatic interaction detection
COPD: chronic obstructive pulmonary disease
CVD: cardiovascular disease
DAG: direct acyclic graph
EPR: electronic patient record
FEV1: forced expiratory volume in one second
FVC: forced vital capacity
GP: general practitioner
ICD-10: International Classification of Diseases 10th Edition
IRR: incidence rate ratio
SES: socioeconomic status
T2DM: type 2 diabetes mellitus
