# Supplementary material for: Digital Recruitment and Acceptance of a Stepwise Model to Prevent Chronic Disease in the Danish Primary Care Sector: Cross-Sectional Study
Source: J Med Internet Res. 2019 Jan 21;21(1):e11658. doi: 10.2196/11658 (PMC6360391; doi:10.2196/11658)
Supplement: Multimedia Appendix 1 [file jmir_v21i1e11658_app1.pdf]

Multimedia appendix 1 – Invitations to consent (first invitation) and to receive a personal digital health profile (second invitation).

Page 2 – Invitation to consent (first invitation)

Page 3 – Invitation for a personal digital health profile (second invitation)

{0}
{1}

{2}

Du har nu mulighed for at få en personlig sundhedsprofil

Sundhedsprofilen giver dig et overblik over din sundhedstilstand og de muligheder du har for at forbedre din livsstil. Du tilbydes også et forløb hos egen læge eller hos din kommune, hvis der er behov for det.

Sådan får du din personlige sundhedsprofil

Du tilmelder dig projektet Tidlig Opsporing og Forebyggelse ved at

1. trykke på knappen nedenfor
2. logge ind med dit nem ID
3. give dit samtykke til, at vi må bruge de sundhedsoplysninger, din egen læge har om dig i sit patientjournalsystem. Oplysningerne fortæller os, om du allerede er i et behandlingsforløb for en kronisk sygdom.

Når du har samtykket, vil vi invitere dig til at udfylde et spørgeskema, som handler om din livsstil og dine sundhedsvaner. På baggrund af dine svar får du den personlige sundhedsprofil, som også indeholder vores anbefalinger til, hvordan du kan forbedre nogle af dine vaner, hvis der er behov for det.

Bemærk venligst, at selvom du samtykker nu, så kommer invitationen til spørgeskemaet først til september i din digitale postkasse. Ventetiden bruger vi til at indhente de oplysninger, der skal bruges til sundhedsprofilen.

Få mere at vide om projektet her: www.projekttof.dk
eller kontakt os på telefon 23 36 24 68

Jeg vil gerne deltage

Hvis knappen ikke virker, kan du gå ind på www.sundhedsmappe.dk og logge ind med dit nemID

Din deltagelse er vigtig

Du modtager dette brev, fordi du er født i perioden 1957-1986. Projekt *Tidlig Opsporing og Forebyggelse* er et forsknings- og samarbejdsprojekt, så samtidig med, at du får en personlig sundhedsprofil bidrager du med værdifuld viden om, hvordan praktiserende læger og kommuner bedst muligt kan tilbyde målrettede forebyggelsesforløb til de borgere, der har behov for det.

Vi opfordrer dig til at deltage, uanset om du er sundog rask, eller om du allerede er i et behandlingsforløb. Hører vi ikke fra dig inden 7 dage, kontakter vi dig igen.

Med venlig hilsen

{3}
{4}

Det med småt

Gennemførelsen af Tidlig Opsporing og Forebyggelse samt vores håndtering af data overholder sundhedsloven og er godkendt af Datatilsynet (Journalnummer: 2015-57-0008).
Vi beklager, hvis du oplever vores henvendelse som utidig eller på anden måde krænkende. I så fald beder vi dig om at se bort fra den. Du kan eventuelt trykke på knappen "Jeg vil gerne deltage" og aktivt anmode om ikke at blive kontaktet yderligere.

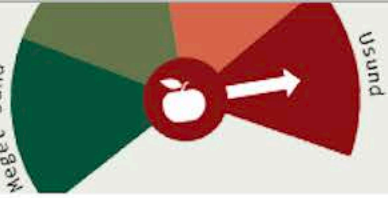

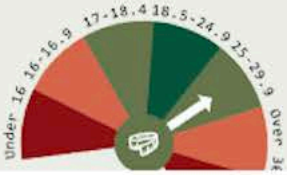

{0}
{1}

{2}

Du har nu mulighed for at få din personlige sundhedsprofil

I april modtog du et brev i din digitale postkasse med en invitation til at deltage i projekt Tidlig Opsporing og Forebyggelse. Du har nu mulighed for at få din personlige sundhedsprofil.

Sådan får du din personlige sundhedsprofil

Nu kan du besvare det spørgeskema, som handler om din livsstil og dine sundhedsvaner. På baggrund af dine svar får du den personlige sundhedsprofil, som også indeholder vores anbefalinger til, hvordan du kan forbedre nogle af dine vaner, hvis der er behov for det. Du tilbydes også et forløb hos egen læge eller hos din kommune, hvis der er behov for det.

Du får adgang til din personlige sundhedsprofil ved at

1. trykke på knappen nedenfor
2. logge ind med dit nem ID
3. besvare spørgeskemaet

Få mere at vide om projektet her: www.projekttof.dk
eller kontakt os på telefon 23 36 24 68

FÅ DIN PERSONLIGE SUNDHEDSPROFIL

Din deltagelse er vigtig

Du modtager dette brev, fordi du er født i perioden 1957-1986 og bosat i Varde/Haderslev kommune. Projekt Tidlig Opsporing og Forebyggelse er et forsknings- og samarbejdsprojekt, så samtidig med, at du får en personlig sundhedsprofil, bidrager du med værdifuld viden om, hvordan praktiserende læger og kommuner bedst muligt kan tilbyde målrettede forebyggelsesforløb til de borgere, der har behov for det.

Vi opfordrer dig til at deltage, uanset om du er sund og rask eller om du allerede er i et behandlingsforløb. Hører vi ikke fra dig inden 7 dage, kontakter vi dig igen.

På forhånd tak for din deltagelse.

Med venlig hilsen

{3}
{4}

Det med småt

Gennemførelsen af Tidlig Opsporing og Forebyggelse samt vores håndtering af data overholder sundhedsloven og er godkendt af Datatilsynet (Journalnummer: 2015-57-0008).
Vi beklager, hvis du oplever vores henvendelse som utidig eller på anden måde krænkende. I så fald beder vi dig om at se bort fra den. Du kan eventuelt trykke på knappen "Jeg vil gerne deltage" og aktivt anmode om ikke at blive kontaktet yderligere.
