# Supplementary material for: Digital Recruitment and Acceptance of a Stepwise Model to Prevent Chronic Disease in the Danish Primary Care Sector: Cross-Sectional Study
Source: J Med Internet Res. 2019 Jan 21;21(1):e11658. doi: 10.2196/11658 (PMC6360391; doi:10.2196/11658)

Multimedia appendix 2 – Data on the acceptance of the second digital invitation in the entire study population (N=8814)

Descriptive analysis of the determinants of the acceptance of the second digital invitation

Determinants	Health profile (%)	No health profile (%)	Total (%)	Missing (%)
**Total**	2,661 (30.19)	6,153 (69.81)	8,814 (100)	
**Demography[a]**				
**Age**				0
29-39	473 (17.78)	2,180 (35.43)	2,653 (30.10)	
40-49	834 (31.34)	2,192 (35.62)	3,026 (34.33)	
50-60	1,354 (50.88)	1,781 (28.95)	3,135 (35.57)	
**Sex**				0
Male	1,164 (43.74)	3,271 (53.16)	4,379 (49.68)	
Female	1,497 (56.26)	2,882 (46.84)	4,435 (50.32)	
**Country of origin**				18 (0.20)
Denmark	2,567 (96.47)	5,264 (85.80)	7,831 (89.03)	
Western	59 (2.22)	490 (7.99)	549 (6.24)	
Non-western	35 (1.32)	381 (6.21)	416 (4.73)	
**Cohabitation**				18 (0.20)
Single	521 (19.58)	1,721 (28.05)	2,242 (25.49)	
Cohabiting	2,140 (80.42)	4,414 (71.95)	6,554 (74.51)	
**Partner in project**			8796	18 (0.20)
Yes	1,525 (57.31)	3,227 (52.60)	4,752 (54.02)	
No	1,136 (42.69)	2,908 (47.40)	4,044 (45.98)	
**Socio-economy[a]**				
**Educational attainment**				583 (6.61)
Secondary school	365 (13.88)	1,342 (23.96)	1,707 (20.74)	
High school	108 (4.11)	248 (4.43)	356 (4.33)	
Vocational education	1,193 (45.38)	2,610 (46.59)	3,803 (46.20)	
Higher education	963 (36.63)	1,402 (25.03)	2,365 (28.73)	
**Employment status**				105(1.19)
Employed	2,212 (83.19)	4,398 (72.69)	6,610 (75.90)	
Self-employed	132 (4.96)	298 (4.93)	430 (4.94)	
Benefits	53 (1.99)	219 (3.62)	272 (3.12)	
Social welfare	228 (8.57)	918 (15.17)	1,146 (13.16)	
Other	34 (1.28)	217 (3.59)	251 (2.88)	
**Family income**		6027	8684	130 (1.47)
Low	351 (13.21)	1,696 (28.14)	2,047 (23.57)	
Middle-low	605 (22.77)	1,542 (25.58)	2,147 (24.72)	
Middle-high	736 (27.70)	1,499 (24.87)	2,235 (25.74)	
High	965 (36.32)	1,290 (21.40)	2,255 (25.97)	
**Medical treatment[b]**				
**Prescriptions and diagnoses[4]**				0
Treatment	588 (22.10)	1,148 (18.66)	1,736 (19.70)	
No treatment	2,073 (77.90)	5,005 (81.34)	7,078 (80.30)	
**Health care usage[c]**				

**Attendance to GP**				0
Yes	2,364 (88.84)	5,181 (84.20)	7,545 (85.60)	
No	297 (11.16)	972 (15.80)	1269 (14.40)	
**Frequent attender**				0
Yes	266 (10.00)	686 (11.15)	952 (10.80)	
No	2,395 (90.00)	5,467 (88.85)	7,862 (89.20)	
**Laboratory tests at GP**				0
Yes	1,557 (58.51)	2,967 (48.22)	4,524 (51.33)	
No	1,104 (41.49)	3,186 (51.78)	4,290 (48.67)	
**Preventive consultation at GP**				0
Yes	336 (12.63)	611 (9.93)	947 (10.74)	
No	2,325 (87.37)	5,542 (90.07)	7,867 (89.26)	

a Social registries and project data
b ATC codes and ICD-10 codes related to type 2 diabetes, cardiovascular disease and chronic obstructive pulmonary disease
c Administrative codes from the general practitioner

Table 2 – Analysis of associations between acceptance of the second digital invitation and socio-demographic determinants, medical treatment, and health care usage in the entire study population

**Determinants**		**Model 1 (Crude)**		**Model 2 (Adjusted for age and sex)**		**Model 3 (Minimally adjusted)**	
	Sample size (N)	IRR[95 % CI]	P-value	IRR [95 % CI]	P-value		P-value
**Age**[b]							
29-39	2,653	1 [0]		1 [0]		1 [0]	
40-49	3,026	1.08 [1.06;1.10]	.001	1.08 [1.06;1.10]	.001	1.08 [1.06;1.10]	.001
50-60	3,135	1.22 [1.19;1.24]	.001	1.21 [1.19;1.24]	.001	1.22 [1.19;1.24]	.001
**Sex**[b]							
Female	4,435	1 [0]		1 [0]		1 [0]	
Male	4,379	0.94 [0.93;0.95]	.001	0.94 [0.93;0.96]	.001	0.94 [0.93;0.95]	.001
**Country of origin**[1b]							
Denmark	7,831	1 [0]		1 [0]		1 [0]	
Western	549	0.83 [0.81;0.85]	.001	0.86 [0.84;0.88]	.001	0.83 [0.81;0.85]	.001
Non-western	416	0.82 [0.80;0.84]	.001	0.84 [0.82;0.86]	.001	0.82 [0.80;0.84]	.001
**Cohabitation**[b]							
Single	2,242	1 [0]		1 [0]		1 [0]	
Cohabiting	6,554	1.08 [1.06;1.09]	.001	1.06 [1.05;1.08]	.001	1.05 [1.03;1.06]	.001
**Partner in project**[c]							
Yes	4,752	1 [0]		1 [0]		1 [0]	
No	4,044	0.97 [0.96;0.98]	.001	0.97 [0.96;0.98]	.001	1.02 [1.00;1.04]	.12
**Educational attainment**[d]							
Secondary school	1,707	1 [0]		1 [0]		1 [0]	
High school	356	1.07 [1.03;1.12]	.001	1.08 [1.04;1.13]	.001	1.09 [1.05;1.13]	.001
Vocational education	3,803	1.08 [1.06;1.10]	.001	1.09 [1.07;1.11]	.001	1.09 [1.07;1.11]	.001
Higher education	2,365	1.16 [1.13;1.18]	.001	1.16 [1.14;1.19]	.001	1.16 [1.14;1.18]	.001
**Employment status**[b]							
Employed	6,610	1 [0]		1 [0]		1 [0]	
Self-employed	430	0.98 [0.95;1.01]	.23	0.97 [0.94;1.00]	.09	0.98 [0.94;1.01]	.14
Benefits	272	0.90 [0.86;0.93]	.001	0.90 [0.87;0.94]	.001	0.93 [0.89;0.97]	.001
Social welfare	1,146	0.90 [0.88;0.92]	.001	0.89 [0.88;0.91]	.001	0.93 [0.91;0.96]	.001
Other	251	0.85 [0.82;0.88]	.001	0.86 [0.83;0.90]	.001	0.89 [0.89;0.99]	.02

**Family income[b]**							
Low	2,047	1 [0]		1 [0]		1 [0]	
Middle-low	2,147	1.09 [1.07;1.12]	.001	1.09 [1.07;1.11]	.001	1.06 [1.04;1.08]	.001
Middle-high	2,235	1.13 [1.11;1.16]	.001	1.11 [1.09;1.14]	.001	1.07 [1.05;1.09]	.001
High	2,255	1.22 [1.20;1.24]	.001	1.16 [1.14;1.18]	.001	1.08 [1.08;1.13]	.001
**Prescriptions and diagnoses[b]**							
Treatment	1,736	1 [0]		1 [0]		1 [0]	
No treatment	7,078	0.97 [0.95;0.98]	.001	1.02 [1.00;1.04]	.02	1.02 [1.00;1.04]	.04
**Attendance at GP[b]**							
Yes	7,545	1 [0]		1 [0]		1 [0]	
No	1,269	0.94 [0.92;0.96]	.001	0.96 [0.94;0.98]	.001	0.98 [0.96;01.00]	.06
**Frequent attender to GP[b]**							
Yes	952	1 [0]		1 [0]		1 [0]	
No	7,862	1.02 [1.00;1.04]	.11	1.04 [1.02;1.06]	.001	1.03 [1.00;1.05]	.03
**Laboratory tests at GP[b]**							
Yes	4,524	1 [0]		1 [0]		1 [0]	
No	4,290	0.94 [0.92;0.95]	.001	0.96 [0.95;0.98]	.001	0.96 [0.95;0.97]	.001
**Preventive consultation at GP[b]**							
Yes	947	1 [0]		1 [0]		1 [0]	
No	7,867	0.96 [0.93;0.98]	.001	1.00 [0.98;1.03]	.94	0.99 [0.97;1.02]	.63

Model 3 adjustments
a No adjustments
b Age, sex, country of origin, education
c Cohabitation
d Age, sex, country of origin

Figure 1 – CHAID analysis of the uptake of the second digital invitation in the entire study population

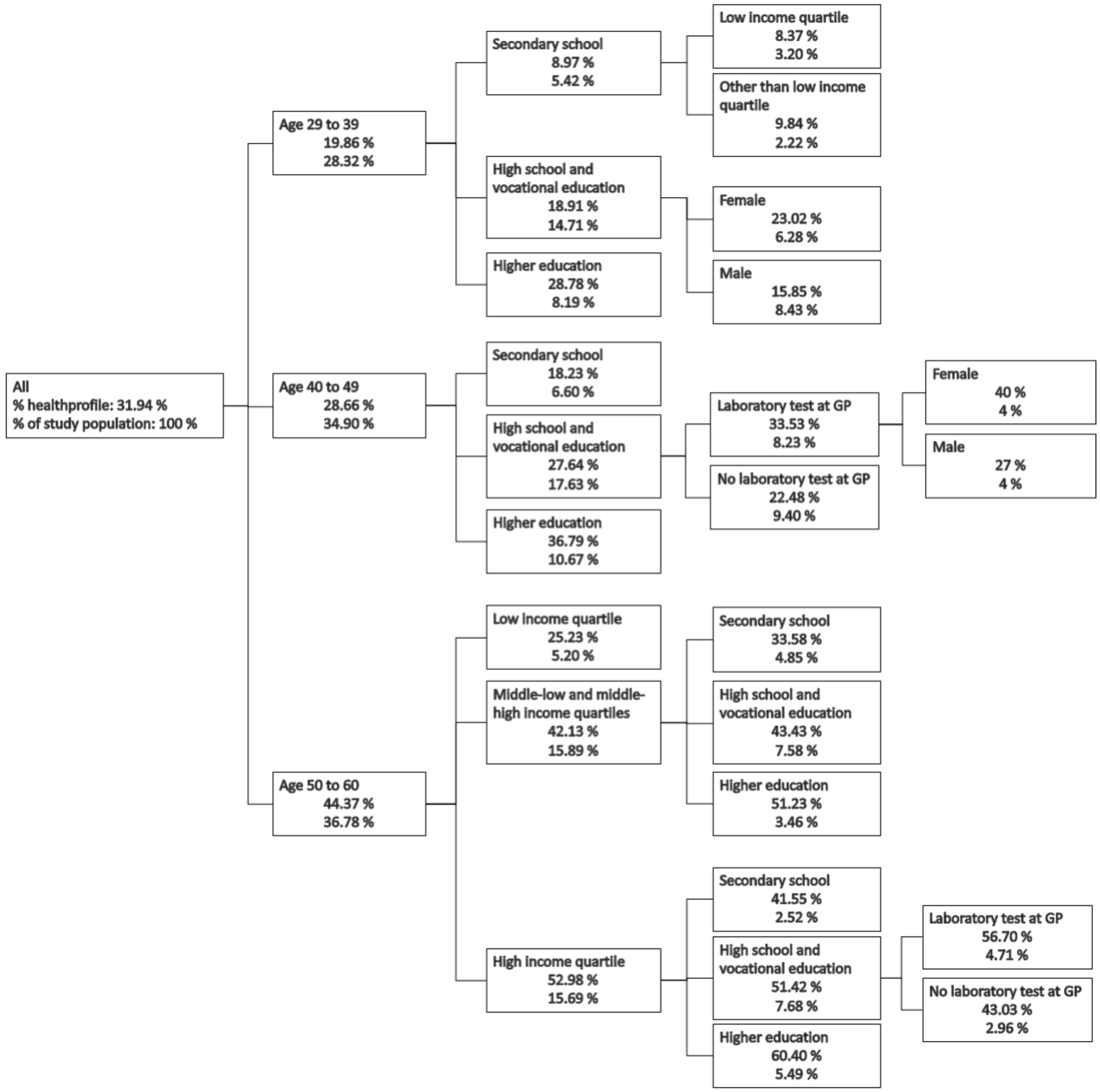

Supplement: Multimedia Appendix 2 [file jmir_v21i1e11658_app2.pdf]
