# Supplementary material for: Digital Recruitment and Acceptance of a Stepwise Model to Prevent Chronic Disease in the Danish Primary Care Sector: Cross-Sectional Study
Source: J Med Internet Res. 2019 Jan 21;21(1):e11658. doi: 10.2196/11658 (PMC6360391; doi:10.2196/11658)

Multimedia appendix 3 – Data on the acceptance of the second digital invitation among those who accepted the first digital invitation (N=3545)

Descriptive analysis of the determinants of the acceptance of the second digital invitation among those who accepted the first digital invitation

Determinants	Health profile (%)	No health profile (%)	Total (%)	Missing (%)
**Total**	2,658 (74.98)[a]	887 (25.02)	3,545 (100)	
**Socio-demography[b]**				
**Age**				0
29-39	471 (17.72)	261 (29.43)	732 (20.65)	
40-49	833 (31.34)	318 (35.86)	1,151 (32.47)	
50-60	1,354 (50.94)	308 (34.72)	1,662 (46.88)	
**Sex**				0
Male	1,163 (43.75)	427 (48.14)	1,590 (44.85)	
Female	1,495 (56.25)	460 (51.86)	1,955 (55.15)	
**Country of origin**				0
Denmark	2,564 (96.46)	821 (92.56)	3,385 (95.49)	
Western	59 (2.22)	32 (3.61)	91 (2.57)	
Non-western	35 (1.32)	34 (3.83)	69 (1.95)	
**Cohabitation**				0
Single	521 (19.60)	205 (23.11)	726 (20.48)	
Cohabiting	2,137 (80.40)	682 (76.89)	2,819 (79.52)	
**Partner in project**				0
Yes	1,523 (57.30)	514 (57.95)	2,037 (57.46)	
No	1,135 (42.70)	373 (42.05)	1,508 (42.54)	
**Educational attainment**				69 (1.95)
Secondary school	365 (13.90)	148 (17.41)	513 (14.76)	
High school	108 (4.11)	35 (4.12)	143 (4.11)	
Vocational education	1,191 (45.35)	413 (48.59)	1,604 (46.14)	
Higher education	962 (36.63)	254 (29.88)	1,216 (34.98)	
**Employment status**				5(0.1)
Employed	2,210 (83.21)	681 (77.04)	2,891 (81.67)	
Self-employed	132 (4.97)	38 (4.30)	170 (4.80)	
Benefits	52 (1.96)	36 (4.07)	88 (2.49)	
Social welfare	228 (8.58)	112 (12.67)	340(9.60)	
Other	34 (1.28)	17 (1.92)	51 (1.44)	
**Family income**			3537	8 (0.2)
Low	351 (13.22)	208 (23.56)	559 (15.80)	
Middle-low	605 (22.78)	203 (22.99)	806 (22.79)	
Middle-high	736 (27.71)	253 (28.65)	989 (27.96)	
High	964 (36.30)	219 (24.80)	1,183 (33.45)	
**Medical treatment[c]**				
**Prescriptions and diagnoses**				0
Treatment	587 (22.08)	176 (19.84)	763 (21.52)	
No treatment	2,071 (77.92)	711 (80.16)	2,782 (78.48)	
**Health care usage[d]**				

**Attendance at GP**				0
Yes	2,361 (88.83)	812 (91.54)	3,173 (89.51)	
No	297 (11.17)	75 (8.46)	372 (10.49)	
**Frequent attender**				0
Yes	264 (9.93)	104 (11.72)	368 (10.38)	
No	2,394 (90.06)	783 (88.28)	3,177 (89.62)	
**Laboratory tests at GP**				0
Yes	1,555 (58.50)	498 (56.14)	2,053 (57.91)	
No	1,103 (41.50)	389 (43.86)	1,492 (42.09)	
**Preventive consultation at GP**				0
Yes	336 (12.64)	95 (10.71)	431 (12.16)	
No	2,322 (87.36)	792 (89.29)	3,114 (87.84)	
**Health checks**				0
….Yes	832 (31.30)	243 (27.40)	1,075 (30.32)	
….No	1,826 (68.70)	644 (72.60)	2,470 (69.68)	

a Three patients withdrew their consent during the intervention (September through December 2017). The withdrawal of the consent changed their status in the database and reduced the total number of patients who accepted the second invitation from 2,661 to 2,658. The difference have no impact on the results

b Social registries and project data

c ATC codes and ICD-10 codes related to type 2 diabetes, cardiovascular disease and chronic obstructive pulmonary disease

d Administrative codes from the general practitioner

Table 2 – Analysis of associations between acceptance of the second digital invitation and socio-demographic determinants, medical treatment, and health care usage among those who accepted the first digital invitation

**Determinants**	Sample size (N)	**Model 1 (Crude)** IRR [95 % CI]	p-value	**Model 2 (Adjusted for age and sex)** IRR [95 % CI]	p-value	**Model 3 (Minimally adjusted)** IRR [95 % CI]	p-value
**Age**[a]							
29-39	732	1 [0]		1 [0]		1 [0]	
40-49	1,151	1.05 [1.02;1.08]	.001	1.05 [1.02;1.08]	.001	1.05 [1.02;1.08]	.001
50-60	1,662	1.10 [1.08;1.13]	.001	1.10 [1.08;1.13]	.001	1.10 [1.08;1.13]	.001
**Sex**[a]							
Female	1,955	1 [0]		1 [0]		1 [0]	
Male	1,590	0.98 [0.97;1.00]	.02	0.98 [0.96;1.00]	.02	0.98[0.97;1.00]	.02
**Country of origin**[a]							
Denmark	3,385	1 [0]		1 [0]		1 [0]	
Western	91	0.94 [0.88;1.00]	.04	0.94 [0.89;.00]	.06	0.94 [0.88;1.00]	.04
Non-western	69	0.86 [0.79;0.93]	.001	0.87 [0.80;0.94]	.001	0.86 [0.79;0.93]	.001
**Cohabitation**[b]							
Single	726	1 [0]		1 [0]		1 [0]	
Cohabiting	2,819	1.02 [1.00;1.5]	.03	1.02 [1.00;1.04]	.07	1.02 [1.00;1.04]	.13
**Partner in project**[c]							
Yes	2,037	1 [0]		1 [0]		1 [0]	
No	1,508	1.00 [0.99;1.02]	.74	1.00 [0.98;1.02]	.97	1.02 [1.00;1.04]	.03
**Educational attainment**[d]							
Secondary school	513	1 [0]		1 [0]		1 [0]	
High school	143	1.03 [0.98;1.07]	.29	1.04 [0.99;1.08]	.14	1.04 [0.99;1.09]	.11
Vocational education	1,604	1.02 [0.99;1.04]	.18	1.03 [1.00;1.05]	.04	1.03 [1.00;1.05]	.05
Higher education	1,216	1.05 [1.02;1.07]	.001	1.06 [1.03;1.09]	.001	1.06 [1.03;1.08]	.001
**Employment status**[b]							
Employed	2,891	1 [0]		1 [0]		1 [0]	
Self-employed	170	1.01 [0.97;1.04]	.71	1.00 [0.97;1.04]	.88	1.01 [0.97;1.04]	.74
Benefits	88	0.90 [0.84;0.96]	.002	0.92 [0.86;0.98]	.01	0.93 [0.87;0.99]	.02
Social welfare	340	0.95 [0.92;0.98]	.001	0.95 [0.92;0.98]	.001	0.96 [0.93;0.99]	.02
Other	51	0.94 [0.87;1.02]	.15	0.95 [0.88;1.02]	.15	1.02 [0.95;1.09]	.65

**Family income[b]**							
Low	559	1 [0]		1 [0]		1 [0]	
Middle-low	806	1.07 [1.04;1.11]	.001	1.07 [1.04;1.10]	.001	1.06 [1.03;1.09]	.001
Middle-high	989	1.07 [1.04;1.10]	.001	1.06 [1.03;1.09]	.001	1.05 [1.02;1.08]	.001
High	1,183	1.11 [1.08;1.15]	.001	1.09 [1.06;1.12]	.001	1.07 [1.04;1.10]	.001
**Prescriptions and diagnoses[b]**							
Treatment	763	1 [0]		1 [0]		1 [0]	
No treatment	2,782	0.99 [0.97;1.01]	.15	1.01 [0.99;1.03]	.36	1.01 [0.99;1.03]	.50
**Attendance at GP[b]**							
Yes	3,173	1 [0]		1 [0]		1 [0]	
No	372	1.03 [1.01;1.06]	.01	1.04 [1.02;1.07]	.001	1.03 [1.01;1.05]	.01
**Frequent attender to GP[b]**							
Yes	368	1 [0]		1 [0]		1 [0]	
No	3,177	1.02 [0.99;1.05]	.15	1.03 [1.00;1.06]	.05	1.02 [0.99;1.05]	.22
**Laboratory tests at GP[b]**							
Yes	2,053	1 [0]		1 [0]		1 [0]	
No	1,492	0.99 [0.97;1.01]	.22	1.00 [0.98;1.02]	.88	1.00 [0.98;1.01]	.64
**Preventive consultation at GP[b]**							
Yes	431	1 [0]		1 [0]		1 [0]	
No	3,114	0.98 [0.96;1.00]	.11	1.00 [0.97;1.02]	.86	0.99 [0.97;1.02]	.53
**Health Check[b]**							
Yes	1,075	1 [0]		1 [0]		1 [0]	
No	2,470	0.98 [0.96;1.00]	.03	1.00 [0.98;1.01]	.60	0.99 [0.98;1.01]	.37

Model 3 adjustments
a No adjustments
b Age, sex, country of origin, education
c Cohabitation
d Age, sex, country of origin

Figure 1 – CHAID analysis of the uptake of the second digital invitation among those who took up the first digital invitation

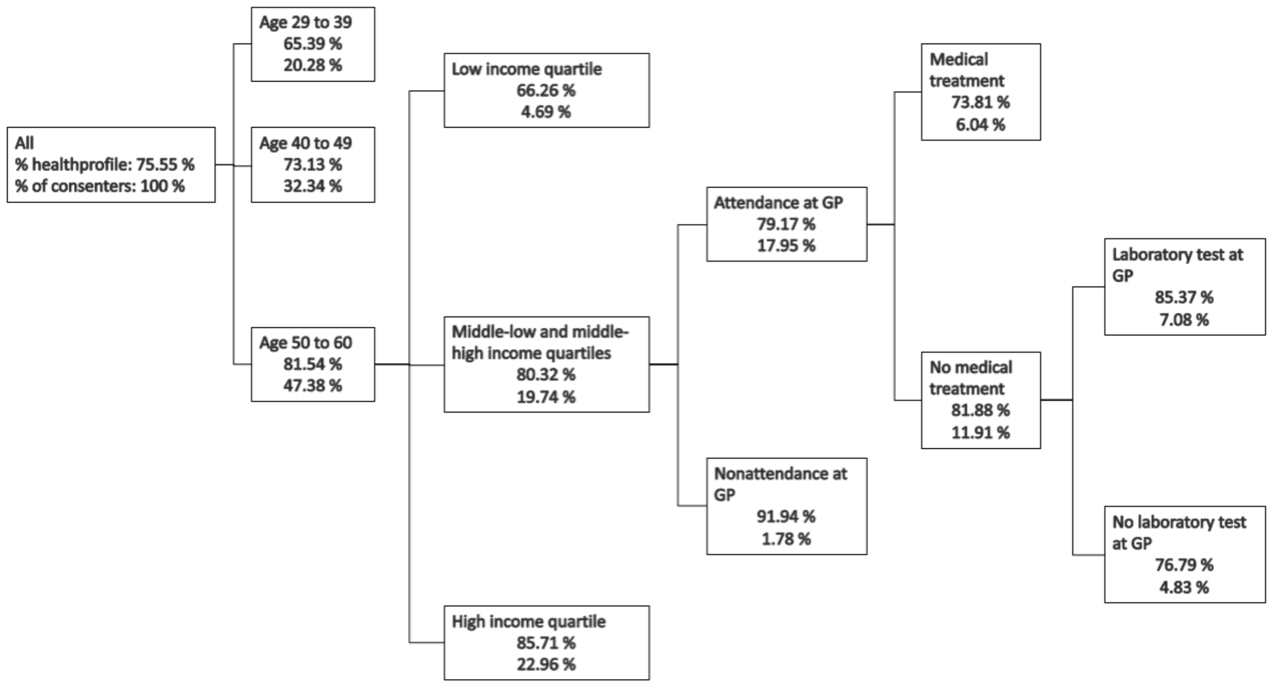

Supplement: Multimedia Appendix 3 [file jmir_v21i1e11658_app3.pdf]
